# Contemporary Outcomes of Infrainguinal Vein Bypass Surgery for Chronic Limb-Threatening Ischaemia: A Two-Centre Cross-Sectional Study

**DOI:** 10.3390/jcm13175343

**Published:** 2024-09-09

**Authors:** Thomas Lovelock, Sharan Randhawa, Cameron Wells, Anastasia Dean, Manar Khashram

**Affiliations:** 1 Department of Vascular Surgery, Waikato Hospital, Hamilton 3204, New Zealand; manar.khashram@gmail.com; 2 Auckland Regional Vascular Service, Auckland City Hospital, Auckland 1023, New Zealand; 3 Faculty of Medical and Health Sciences, The University of Auckland, Auckland 1023, New Zealand

**Keywords:** chronic limb-threatening ischaemia, infrainguinal bypass, Aotearoa New Zealand

## Abstract

**Background/Objectives**: Chronic limb-threatening ischaemia (CLTI) is a significant life and limb-threatening condition. Two recent seminal trials, BEST-CLI and BASIL-2, have provided seemingly conflicting results concerning the optimal treatment modality for patients with CLTI. We sought to investigate the outcomes of patient undergoing infrainguinal bypass at two centres in Aotearoa New Zealand. **Methods**: A cross-sectional retrospective review of all patients who underwent infrainguinal bypass grafting for CLTI at Auckland City Hospital and Waikato Hospital between January 2020 and December 2021 was performed. The primary outcome was a composite of death, above-ankle amputation, and major limb reintervention. The secondary outcome was minor limb reintervention. Kaplan–Meier survival analysis was performed to determine time to the primary and secondary endpoints. Demographic factors were examined using the log-rank test to examine the effect on the outcome. **Results:** One hundred and nineteen patients who underwent infrainguinal bypass for CLTI in the study period were identified. Of these, 93 patients had a bypass with ipsilateral or contralateral GSV. The median follow-up time was 1.85 years. The most common indication for surgery was tissue loss (69%, *n* = 63), with the most common distal bypass target being the below-knee popliteal artery (45%, *n* = 41). The primary composite outcome occurred in 42.8% of the cohort (*n* = 39). Death was the most common component of the primary outcome (26%, *n* = 24). Male sex (HR 0.48, 95% CI 0.26–0.88, *p* = 0.018) and statin use (HR 0.49, 95% CI 0.24–0.98, *p* = 0.044) were independent predictors of protection from the composite outcome on multivariate analysis. Dialysis dependence (HR 3.32, 95% CI 1.23–8.99, *p* = 0.018) was an independent predictor for patients meeting the composite outcome. **Conclusions**: This study’s results are consistent with the published outcomes of BEST-CLI. The patient cohorts examined, anatomical disease patterns, and conduit use may explain some of the differences observed between this study, BEST-CLI and BASIL-2. Further work is required to define the specific patient populations who will benefit most from an open surgical or endovascular first approach to the management of CLTI.

## 1. Introduction

Background/Rationale: Chronic limb-threatening ischaemia (CLTI) is an advanced manifestation of peripheral arterial disease with high rates of limb loss and mortality in the medium term. The treatment paradigm for CLTI is a surgical bypass (commonly using great saphenous vein (GSV)) or endovascular therapy utilising balloon angioplasty with a wide range of adjuncts, including stenting [1,2,3].

Historical data from the BASIL-1 trial suggested that infrainguinal bypass offered superiority with regard to mortality and limb preservation compared to endovascular therapy [1]. Since the seminal trial, however, vast improvements in endovascular technology, particularly drug-eluting technologies, have prompted the re-consideration of clinical equipoise when selecting an optimal treatment modality for patients with CLTI [2]. In 2022 and 2023, the publication of BEST-CLI and BASIL-2 addressed the uncertainty regarding the optimal treatment strategy for CLTI in the modern era. BEST-CLI, a randomised prospective multinational study that included over 1800 patients, compared patients who underwent either infrainguinal bypass (+/− aortoiliac inflow procedure as necessary) or endovascular therapy. The main arm of the trial was a sub-cohort of 1434 patients with adequate GSV (>3 mm) for bypass grafting. The trial concluded that patients with adequate GSV who underwent surgical bypass grafting had a lower incidence of a composite outcome of death, above-ankle amputation, or major limb reintervention (revision or redo surgical bypass grafting, surgical thrombectomy, or thrombolysis) compared to endovascular therapy [3]. In contrast, the BASIL-2 trial, a randomised prospective multinational trial of 345 patients, found increased rates of a composite outcome of death or major amputation in those undergoing infrapopliteal surgical vein bypass with or without concomitant femoropopliteal revascularization when compared to an endovascular first strategy [4].

At a glance, heterogeneity in the trial populations with regard to age, anatomical disease patterns, treatment modalities, and perioperative complication rates, may be responsible for some of the differences observed [5,6,7]. The applicability of this new international data to the Australasian population with CLTI is unclear. Two centres in Aotearoa New Zealand (AoNZ), Auckland City Hospital and Waikato Hospital, have a “bypass first” approach to patients with CLTI, where patients undergo ultrasound mapping of bilateral GSV at the time of index arterial imaging. Those patients with adequate calibre GSV and are deemed fit candidates for surgical bypass are treated preferentially with an open surgical approach.

Objectives: This study aimed to investigate the outcomes of patients treated with infrainguinal vein bypass for CLTI in light of the published outcomes and results of the BEST-CLI trial.

## 2. Materials and Methods

This cross-sectional study was reported using the Strengthening the Reporting of Observational Studies in Epidemiology (STROBE) guidelines [8].

Study design: This was a cross-sectional retrospective review.

Setting: All patients who underwent infrainguinal bypass grafting for CLTI with either ipsilateral or contralateral GSV between January 2020 and December 2021 at either Waikato Hospital or Auckland City Hospital.

Participants: The Australasian Vascular Audit (AVA), a prospective binational surgical audit system used for all vascular surgical procedures in Australia and New Zealand, was used to identify all patients who underwent infrainguinal bypass grafting between the prescribed dates. This was cross-referenced against pre-existing prospective databases maintained at each institution for research purposes. Patients who underwent infrainguinal bypass grafting for aneurysmal disease, trauma, or acute limb ischaemia were excluded. Patients who underwent infrainguinal bypass grafting with a conduit other than a single-segment GSV were also excluded. Ethics approval for this study was granted by the New Zealand Central Health and Disability Ethics Committee (20/CEN/122).

Variables: The primary outcome of the study was a composite outcome of death, above-ankle amputation, or major limb reintervention. This endpoint was chosen as it was the primary outcome of the BEST-CLI trial. Deaths were identified using electronic hospital medical records and the New Zealand National Mortality Database. Above-ankle amputation was defined as an amputation above the ankle joint, most commonly below or above knee amputation. Major limb reintervention was defined as open redo surgical bypass or graft revision, surgical thrombectomy, or endovascular thrombolysis. Secondary outcomes were death, above-ankle amputation, and major limb reintervention (all examined as standalone outcomes), as well as the incidence of minor limb reintervention (defined as endovascular graft angioplasty with or without other adjuncts). The follow-up time period was defined as the time between index surgery and the time point at which the patient last interacted with the health system—either surveillance duplex, the vascular outpatient clinic, or hospitalisation (either under a vascular surgical service or other medical services). Additional data were extracted from hospital medical records, including demographic data (age, gender, ethnicity), comorbidities (smoking status, history of hypertension, ischaemic heart disease, chronic kidney disease (defined as a serum creatinine of greater than 150 μmol/L or dialysis dependence), diabetes mellitus), and perioperative medications (antiplatelet use, anticoagulants, and statins). Indications for surgery were documented (rest pain or tissue loss), and operative details include the site of proximal and distal anastomosis site, distal runoff vessels, conduit choice, and vein configuration. Using a combination of the National Health Identifier, the Australasian Vascular Audit, and hospital electronic medical records, complete data for all patients were obtained.

Data sources/measurement: Data from index admission and operative intervention were obtained from hospital electronic medical records and cross-referenced against data entered into the Australasian Vascular Audit. Follow-up data were obtained from hospital electronic medical records and included both clinical, operative, and imaging surveillance data. Mortality data were obtained from hospital electronic medical records and the New Zealand National Mortality Audit.

Bias: There is selection bias in this study due to the lack of a comparison arm against which to compare the demonstrated infrainguinal bypass graft outcomes. There is also selection bias among the participants in this study as it may not capture patients who were deemed unfit or unsuitable for surgery. The retrospective cross-sectional nature of the study precluded addressing these biases during study design.

Study size: A two-year period was selected to obtain a reasonable, real-world sample size of infrainguinal bypass at two tertiary centres. Patients undergoing infrainguinal bypass after December 2021 were not included due to lesser follow-up time periods.

Quantitative variables: No quantitative variables were examined as an endpoint in this analysis.

Statistical methods: Statistical analysis was performed using RStudio (Version 2022.07.2; R Foundation for Statistical Computing, Vienna, Austria). Continuous data were represented as mean/median (with IQR) and categorical data as a percentage. Simple descriptive statistics were used to tabulate the data. Wilcoxon signed-rank tests were performed to determine statistical differences between sub-groups. Kaplan–Meier survival analysis was performed to assess the time to the composite endpoint, mortality, above-ankle amputation, major limb reintervention, or minor reintervention. The log-rank test was used to assess the effect of various demographic and surgical factors on these outcomes. Multivariate analysis was undertaken using Cox regression, with models constructed using variables with a *p* value of <0.1 on univariate analysis. The chi-square test was used to assess differences between the New Zealand dataset and the data reported in BEST-CLI. A *p* value of less than 0.05 was considered statistically significant.

## 3. Results

**Figure 1:**
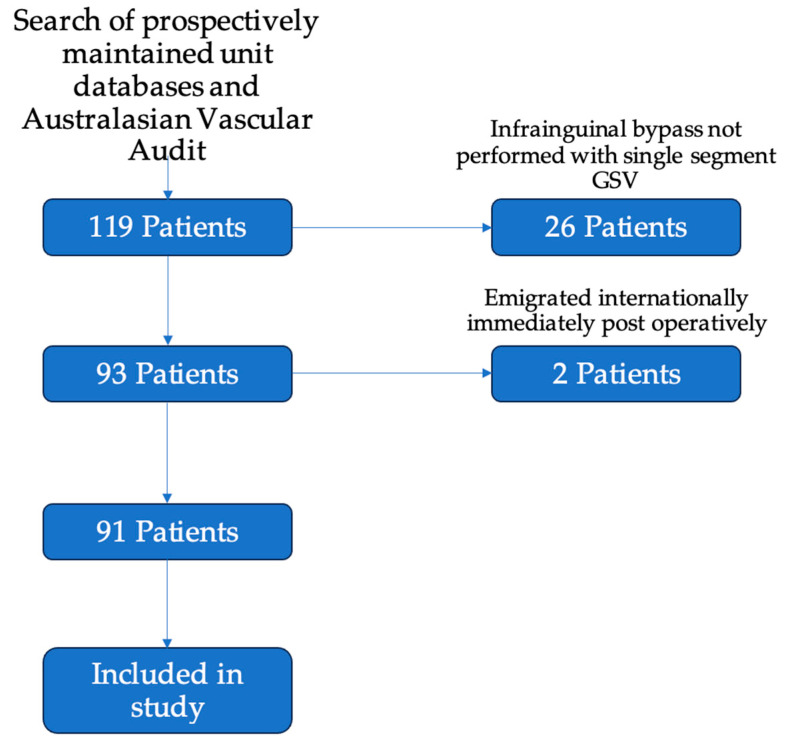
Flowchart of inclusion of patients in the study. Of the included 119 patients, 26 were excluded for having bypasses without single segment GSV—these included 9 bypasses using arm vein, 8 using prosthetic conduit, and 9 patients with spliced or composite conduit. A further 2 patients were excluded as they emigrated from New Zealand immediately after their post-operative period. A total of 91 patients were included for analysis in the study.

Participants: There were 119 patients who underwent infrainguinal bypass grafting over the study period (65 at Auckland City Hospital; 54 at Waikato Hospital). Of these, 93 patients underwent infrainguinal bypass grafting with a single segment of the great saphenous vein. Two patients were lost to early follow-up, with both emigrating to international locations immediately after discharge from their index operation. One was an international patient, and one was a New Zealand citizen who relocated. Ninety-one patients attended at least one post-operative follow-up appointment (Figure 1).

Descriptive data: The AoNZ cohort and the BEST-CLI cohort had similar median age, gender, and ethnicities (Table 1). The prevalence of major vascular comorbidities and perioperative antiplatelet and statin therapy in the two cohorts was also similar (Table 1).

**Figure 2:**
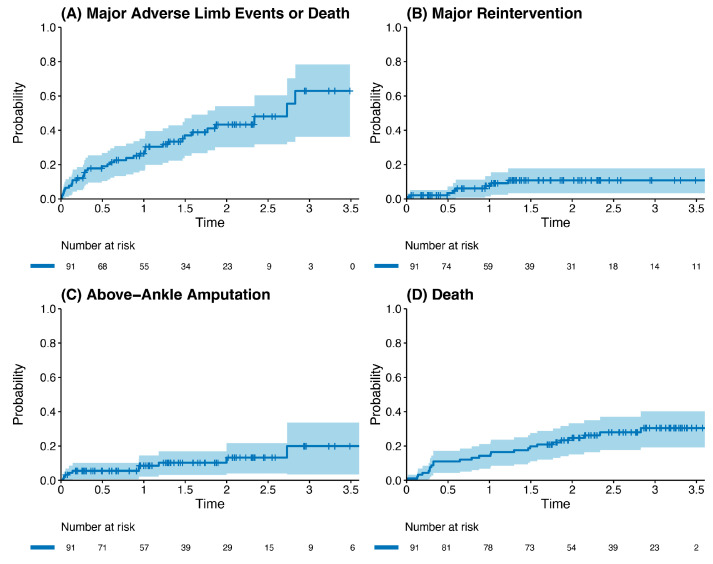
Kaplan–Meier plot of outcomes post infrainguinal bypass grafting (all vein grafts), stratified by incidence of the primary composite outcome (**A**), major reintervention (**B**), above-ankle amputation (**C**), and death (**D**).

**Figure 3:**
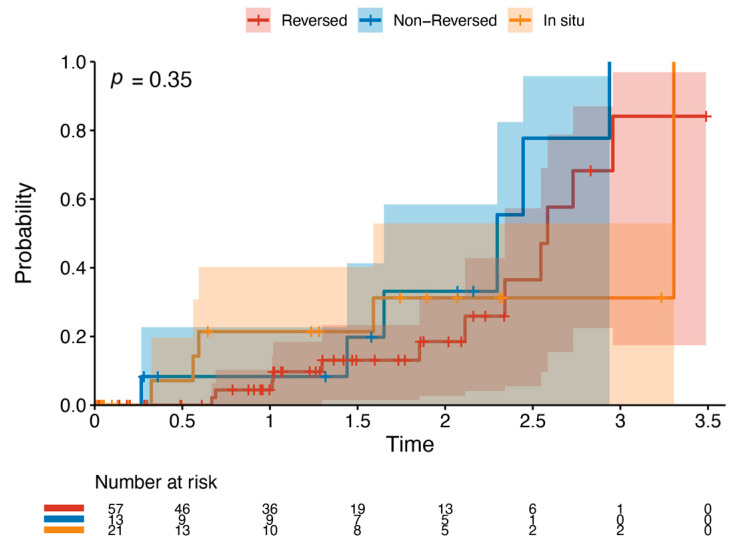
Kaplan–Meier plot of graft angioplasty stratified by vein orientation. This details the likelihood of requiring graft angioplasty by a specific time point when stratifying by vein orientation—reversed (red), non-reversed (blue), or in situ (orange).

Outcome data and main results: The most common indication for intervention was tissue loss (69%, *n* = 63), with the most common bypass target the below-knee popliteal artery (45%, *n* = 41) (Table 2). The median follow-up time was 1.85 years (IQR 1.36–2.33). The primary outcome occurred in 42.8% of the cohort (*n* = 39). Death was the most common endpoint (26%, *n* = 24), followed by major reintervention (9%, *n* = 8) and major amputation (8%, *n* = 7). Four patients who had met the primary outcome by having a major reintervention subsequently went on to have a major amputation (4%, *n* = 4). Three patients who underwent major amputation subsequently died (3%, *n* = 3). These patients died a median of 433 days after the major amputation (Interquartile range (IQR) 266–556.5 days). There was one perioperative death within the index hospital admission (1%, *n* = 1). Figure 2 and Table 3 detail AoNZ cohort outcomes, with reference to reported BEST-CLI outcomes. The secondary outcome (graft angioplasty for bypass graft stenosis) occurred in 22% of patients (*n* = 20) at a mean time of 117 +/− 105 days (mean +/− standard deviation) post-operatively. The impact of differing vein configurations (reversed, non-reversed, and in situ) was analysed with regard to graft angioplasty rates. There were no significant differences in the incidence of graft angioplasty when stratifying grafts according to vein orientation (*p* = 0.35) (Figure 3).

**Figure 4:**
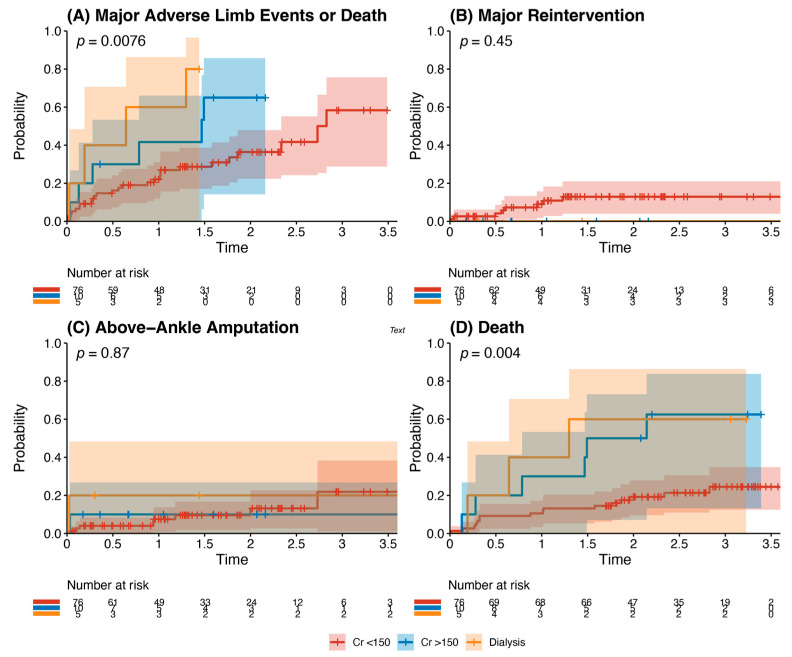
Outcomes of patients with chronic kidney disease after infrainguinal bypass grafting. This details the incidence of the primary composite outcome (panel **A**), major reintervention (panel **B**), above-ankle amputation (Panel **C**), or death (Panel **D**). This is stratified into patients with a creatinine <150 μmol/L (red), with a creatinine >150 μmol/L but not dialysis-dependent (blue), and those who were dialysis-dependent (orange). There was a significantly increased risk of major adverse limb events or death (*p* = 0.0076) and death (*p* = 0.004) in patients with chronic kidney disease undergoing infrainguinal bypass grafting for chronic limb-threatening ischaemia.

Other analyses: Kaplan–Meier analysis of age and recorded risk factors was performed to identify predictors of the composite outcome. Chronic kidney disease (baseline serum creatinine >150 μmol/L) and end-stage renal failure requiring dialysis (*p* = 0.0076) were statistically significant predictors of the composite outcome, with significantly higher rates of death (*p* = 0.004) (Figure 4). There were no statistically significant differences in outcomes for patients when stratified by age >65 years, history of hypertension, history of ischaemic heart disease, history of diabetes mellitus, and smoking history (*p* all > 0.05). Multivariate analysis demonstrated male sex (HR 0.48, 95% CI 0.26–0.88, *p* = 0.018) and statin use (HR 0.49, 95% CI 0.24–0.98, *p* = 0.044) as independent risk factors that reduced the risk of the composite outcome. Dialysis dependence (HR 3.32, 95% CI 1.23–8.99, *p* = 0.018) was an independent risk factor for the composite outcome (Table 4).

## 4. Discussion

Key results: This study reports medium-term outcomes of infrainguinal vein bypass for CLTI from two centres in Aotearoa New Zealand. Our study demonstrated remarkably similar data when compared to the data reported from BEST-CLI with regard to perioperative demographics, risk factors, and outcomes despite the nature of study populations. Forty-three percent of patients in our study and the BEST-CLI trial met the composite outcome [3]. The most common component of the composite outcome met was death (26% in the AoNZ cohort vs. 33% in BEST-CLI). Major amputation, major reintervention, and perioperative death rates were all similar (12.1% vs. 10.4%; 8.8% vs. 9.2%; 1.1% vs. 1.7%) [3].

Interpretation and generalisability: Our study outcomes align with the BEST-CLI trial and conflict with the findings of BASIL-2, the other major contemporaneously published lower limb CLTI trial [3,4]. Several other recent international retrospective studies have echoed improved wound healing and amputation-free survival in patients treated with infrainguinal bypass over endovascular intervention for CLTI patients [9,10]. The differences between the patient populations in BEST-CLI and BASIL 2 have been somewhat elucidated, although further collaboration between the principal investigators of each trial is expected to yield greater clarification on this. Both trials did not meet their recruitment targets, primarily as an effect of the COVID-19 pandemic. Patients in BASIL-2 were significantly older than in BEST-CLI and the AoNZ cohort, with a median age of 72 years, compared to 67 years (BEST-CLI) and 69 years (AoNZ cohort), respectively. Moreover, enrolment in BASIL-2 required a life expectancy of only 6 months compared to a requirement of a life expectancy of 2 years to be included in BEST-CLI [4,5]. The European Society for Vascular Surgery (ESVS) Clinical Practice Guidelines define an “average risk” surgical patient as having a >50% 2-year life expectancy, with a perioperative mortality risk of <5% (Evidence Grade 2C) [11]. Taking into account simultaneous limb staging and the anatomical pattern of disease, ESVS guidelines recommend consideration of endovascular revascularization as the primary strategy in “high risk” patient subgroups that are anatomically suitable for endovascular intervention [11]. In BASIL-2, patients undergoing surgical bypass had a significantly higher rate of previous myocardial infarction (24% vs. 13% in the endovascular arm) [4]. Perioperative mortality after bypass surgery in BASIL-2 was 6%, significantly higher than that in BEST-CLI (1.7%) and our study (1.1%) [3,4]. It is possible that some of the differences between our study and BEST-CLI and BASIL-2 may be explained by patient selection, where patients are deemed not fit for surgical bypass and are either treated with conservative/palliative management or endovascular therapy where possible. This is reinforced by other studies, which have suggested an endovascular strategy to be equivalent to open surgery in high-risk patient groups [12]. Certainly, it is true that the “bypass first” approach at our institutions may mean that higher-risk patients deemed unsuitable for bypass may not be represented, which may reduce the generalizability of our results to higher-risk cohorts.

Differences in the nature of the conduit used may account for some of the observed differences. In our cohort, all of the included patients had suitable contralateral or ipsilateral GSV for bypass grafting. The primary cohort of BEST-CLI included patients with an adequate single segment of GSV for infrainguinal bypass grafting, whereas BASIL-2 included patients who had any segment of vein deemed suitable for bypass by a vascular surgeon. In total, 79% of patients in BASIL-2 had bypasses with either ipsilateral or contralateral GSV [4]. BEST-CLI’s secondary cohort compared patients without an adequate single-segment GSV to endovascular therapy, with 29% using alternative autogenous conduit, such as an arm vein. This cohort demonstrated no significant difference between endovascular therapy and surgical bypass [3]. Heterogeneity in the trial populations with regard to choice of conduit, as well as other local factors, such as the quality of pre-operative ultrasound mapping and surgeon assessment of the suitability of the conduit, may account for some of the differences seen between our study and the two landmark trials.

The anatomical pattern of peripheral arterial disease may also contribute to the observed differences. In our study, 66% of the bypasses were anastomosed distally to the popliteal artery, with only 7% to a distal tibial or pedal target. Similarly, in BEST-CLI, 44% of patients underwent femoropopliteal bypass, and 40% underwent femorotibial bypass [3]. Fifty two percent of bypasses in BASIL 2 were to a distal tibial or pedal target, with forty percent originating from the popliteal artery [4]. This suggests distinct heterogeneity between the disease patterns being treated between each trial and our current study. In spite of this, a recent subgroup analysis of BEST-CLI suggested a sustained benefit for open surgery over endovascular therapy when solely analysing trial participants with infrapopliteal disease [13]. Other centres have also reported a benefit of a bypass-first strategy for the treatment of infrapopliteal arterial disease [14].

This study found that female sex is an independent risk factor for the composite endpoint of death, major revascularization, or major amputation. It has previously been reported that women with CLTI in AoNZ have worse survival compared to males and higher rates of reintervention and complications in a global cohort [15,16]. Some of our findings may be explained by the higher frequency of smaller target vessels present in a female population, which may render revascularization less durable. It also has been shown that women have lower prescriptions of the best medical therapy following the diagnosis of peripheral arterial disease [17]. Further work is required to elucidate the exact mechanisms by which females with CLTI have lesser outcomes after infrainguinal bypass surgery. This may provide further insight into the best use of infrainguinal bypass grafting vs. endovascular therapy in this cohort of patients.

Limitations: There are several limitations to this study. The non-randomised retrospective observational nature of the study means that there are inherent selection biases. Any patients who underwent endovascular therapy for CLTI (including those who were declined for surgical bypass) have not been captured; therefore, there was no comparison arm for endovascular therapy. Difficulties with patient follow-up, which is typical with an elderly, comorbid population, may influence the accuracy of the data obtained. We included all patients who underwent infrainguinal bypass grafting for CLTI at our institutions, which also encompasses some patients who presented with diabetic foot sepsis. It is possible that some of the amputations reported were as a result of sepsis rather than the failure of the revascularization strategy. The sample size of our cohort is small in comparison to larger European and North American centres, reflecting the realities of vascular surgical centres in Australasia. The small sample size is not well-powered for multivariable analysis. The follow-up period of our study was less (median follow-up 1.85 years) than in BEST-CLI (2.7 years). All patients with CLTI were included, with no stratification for the degree of tissue loss or extent of atherosclerotic disease. While including patients on an all-comer basis does provide some benefit on the generalizability of a bypass-first approach to these patients, stratification of patients into disease patterns or ischaemia severity may have provided further insight into patients who will benefit greatest from an open surgical revascularization strategy.

While BEST-CLI and BASIL-2 have undoubtedly given a greater insight into the treatment paradigm for patients with CLTI, there are other aspects that require further investigation. The time between presentation and intervention has long been thought to influence outcomes [18]. BEST-CLI patients waited a median of 1 and 4 days before intervention for endovascular therapy and bypass, respectively [3]. In BASIL-2, 82% of endovascular and 80% of bypass patients were treated within 2 weeks [4]. We have no data regarding the time to treatment in this cohort. This is particularly pertinent in the Australasian setting, where timely access to quality duplex sonography, angiography suites, and operating theatres may differ between institutions, and there are often large geographic distances between patient populations and vascular surgical referral centres. Health-related quality of life and cost considerations are increasingly important metrics in an Australasian setting with increasing healthcare costs and an ageing population, and something for which there is scarce data in this context [19,20].

## 5. Conclusions

Mid-term outcomes of infrainguinal bypass grafting for patients CLTI in Aotearoa New Zealand appear comparable to those reported in the BEST-CLI trial. More work is required to elucidate the specific patient populations who will benefit most from an open surgical approach, and those in whom endovascular therapy as a primary strategy is best.

## Figures and Tables

**Table 1:** Patient demographics, comorbidities, and perioperative medications.

	Aotearoa New Zealand	BEST-CLI	*p*-Value
PATIENTS	91	718	
AGE (YEARS)	68.8 +/− 11.6 ^	66.9 +/− 9.8 ^	
FEMALE SEX	25 (27%)	201 (28%)	*p* = 0.94
ETHNICITY			
CAUCASIAN	65 (71%)	500 (70%)	
MAORI	13 (14%)		
BLACK		156 (22%)	
OTHER	11 (12%)	55 (8%)	
SMOKING STATUS			
EX	38 (42%)	Not reported	
CURRENT	35 (38%)	264 (37%)	
HTN	80 (88%)	620 (87%)	*p* = 0.91
IHD	44 (48%)	301 (42%)	*p* = 0.47
CKD			
CREATININE >150 µMOL/L	10 (11%)	Not reported	
ON DIALYSIS	5 (5%)	67 (9%) *	
DIABETES MELLITUS			
T1DM	4 (4%)	Not reported	
T2DM	36 (40%)	513 (72%)	
ANTIPLATELET	83 (91%)	619 (87%)	*p* = 0.73
ANTICOAGULANT	26 (29%)	73 (10%)	*p* < 0.0001
STATIN	75 (82%)	503 (70%)	*p* = 0.33

* Reported as ESRF in the BEST-CLI trial, ^ Standard deviation.

**Table 2:** Operative details.

INDICATION	
REST PAIN	28 (31%)
TISSUE LOSS	63 (69%)
PROXIMAL ANASTOMOSIS	
CFA	48 (53%)
SFA/PFA	39 (43%)
POPLITEAL	3 (3%)
OTHER	1 (1%)
DISTAL TARGET	
ABOVE KNEE POPLITEAL	19 (21%)
BELOW KNEE POPLITEAL	41 (45%)
TIBIAL	25 (27%)
DISTAL TIBIAL/PEDAL	6 (7%)
CONDUIT	
IPSILATERAL GSV	86 (95%)
CONTRALATERAL GSV	5 (5%)

**Table 3:** Outcomes after infrainguinal bypass for CLTI.

	AOTEAROA NEW ZEALAND	BEST CLI	*p*-Value
MEDIAN FOLLOW-UP	1.85 years (IQR 1.4–2.3)	2.7 years (IQR 1.6–4.0)	
PRIMARY OUTCOME	39 (42.8%)	302 (42.6%)	
DEATH *	27 (30%)	234 (33%)	*p* = 0.68
MAJOR AMPUTATION ^	11 (12%)	74 (10.4%)	*p* = 0.64
MAJOR REINTERVENTION	8 (9%)	65 (9.2%)	*p* = 0.94
PERIOPERATIVE DEATH	1 (1%)	12 (1.7%)	*p* = 0.69

IQR: Interquartile range. * 24 patients died without prior major amputation or major reintervention. A further 3 patients died after major amputation. ^ 7 patients had a major amputation without prior major reintervention. A further 4 patients underwent major amputation subsequent to a major reintervention.

**Table 4:** Multivariate analysis of factors leading to the composite outcome.

	UNIVARIATE (HR (95% CI, *p*-Value))	MULTIVARIATE (HR, (95% CI, *p*-Value))
AGE	1.01 (0.99–1.04, *p* = 0.263)	-
GENDER (FEMALE) (REFERENCE)		
GENDER (MALE)	0.45 (0.25–0.81, *p* = 0.008)	0.48 (0.26–0.88, *p* = 0.018)
ETHNICITY (EUROPEAN) (REFERENCE)		
ETHNICITY (MAORI)	1.39 (0.69–2.81, *p* = 0.358)	
ETHNICITY (OTHER)	1.29 (0.31–5.42, *p* = 0.728)	
SMOKING (NON-SMOKER) (REFERENCE)		
SMOKING (EX-SMOKER)	1.03 (0.45–2.32, *p* = 0.950)	
SMOKING (CURRENT SMOKER)	1.00 (0.43–2.32, *p* = 0.993)	
HYPERTENSION	2.29 (0.71–7.39, *p* = 0.165)	
ISCHAEMIC HEART DISEASE	1.58 (0.89–2.82, *p* = 0.118)	
CHRONIC KIDNEY DISEASE (CREATININE <150 μMOL/L) (REFERENCE)		
CHRONIC KIDNEY DISEASE (CREATININE >150 μMOL/L)	2.38 (1.00–5.67, *p* = 0.051)	1.64 (0.65–4.10, *p* = 0.292)
CHRONIC KIDNEY DISEASE (DIALYSIS-DEPENDENT)	3.17 (1.23–8.18, *p* = 0.017)	3.32 (1.23–8.99, *p* = 0.018)
DIABETES MELLITUS	1.75 (1.00–3.07, *p* = 0.052)	1.52 (0.82–2.80, *p* = 0.182)
ANTIPLATELET USE	1.13 (0.45–2.87, *p* = 0.790)	
ANTICOAGULANT USE	0.71 (0.37–1.36, *p* = 0.299)	
STATIN USE	0.54 (0.28–1.05, *p* = 0.068)	0.49 (0.24–0.98, *p* = 0.044)
INDICATION FOR OPERATION (REST PAIN) (REFERENCE)		
INDICATION FOR OPERATION (TISSUE LOSS)	0.84 (0.45–1.56, *p* = 0.572)	
ORIENTATION OF VEIN (REVERSED) (REFERENCE)		
ORIENTATION OF VEIN (NON REVERSED)	0.38 (0.12–1.25, *p* = 0.112)	
ORIENTATION OF VEIN (IN SITU)	1.51 (0.76–3.01, *p* = 0.242)	
DISTAL RUNOFF (3 CRURAL VESSELS) (REFERENCE)		
DISTAL RUNOFF (2 CRURAL VESSELS)	1.55 (0.83–2.91, *p* = 0.167)	
DISTAL RUNOFF (1 CRURAL VESSEL)	0.81 (0.37–1.78, *p* = 0.605)	

## Data Availability

Data from this study can be provided upon request to the corresponding author.
